# Factors Affecting the Timing of Signal Detection of Adverse Drug Reactions

**DOI:** 10.1371/journal.pone.0144263

**Published:** 2015-12-07

**Authors:** Masayuki Hashiguchi, Shungo Imai, Keiko Uehara, Junya Maruyama, Mikiko Shimizu, Mayumi Mochizuki

**Affiliations:** 1 Division for Evaluation and Analysis of Drug Information, Faculty of Pharmacy, Keio University, Shibakoen, Minato-ku, Tokyo, Japan; 2 Japan Pharmaceutical Information Center (JAPIC), Shibuya, Shibuya-ku, Tokyo, Japan; 3 Department of Hygienic Chemistry, Faculty of Pharmacy, Keio University, Shibakoen, Minato-ku, Tokyo, Japan; Kyushu University, JAPAN

## Abstract

We investigated factors affecting the timing of signal detection by comparing variations in reporting time of known and unknown ADRs after initial drug release in the USA. Data on adverse event reactions (AERs) submitted to U.S. FDA was used. Six ADRs associated with 6 drugs (rosuvastatin, aripiprazole, teriparatide, telithromycin, exenatide, varenicline) were investigated: Changes in the proportional reporting ratio, reporting odds ratio, and information component as indexes of signal detection were followed every 3 months after each drugs release, and the time for detection of signals was investigated. The time for the detection of signal to be detected after drug release in the USA was 2–10 months for known ADRs and 19–44 months for unknown ones. The median lag time for known and unknown ADRs was 99.0–122.5 days and 185.5–306.0 days, respectively. When the FDA released advisory information on rare but potentially serious health risks of an unknown ADR, the time lag to report from the onset of ADRs to the FDA was shorter. This study suggested that one factor affecting signal detection time is whether an ADR was known or unknown at release.

## Introduction

Currently, the early detection of adverse drug reactions (ADRs) caused by drugs that are already on the market is the prime concern of pharmacovigilance efforts. Therefore, both pharmaceutical companies and regulatory authorities have an interest in improving pharmacovigilance methods. The analysis of spontaneous reports of suspected ADRs is a valuable tool [1].

Until now, there are many data mining reports to detect rare and/or unknown ADRs using spontaneous ADRs reporting system [2]. In recent years, not only detecting rare and/or unknown ADRs, spontaneous ADRs reporting system is utilized for identifying hidden drug-drug interactions [3] and potential drug target prediction [4].

Several data-mining methods using large spontaneous reporting databases of ADRs such as the Adverse Event Reporting System (AERS) of the Food and Drug Administration (FDA) in the USA, Eudravigilance in the EU, and VigiBase in the Uppsala Monitoring Centre of the World Health Organization (WHO) are used to detect signals of rare or unknown ADRs in a timely manner. Those databases are becoming increasingly important in pharmacovigilance.

Presently, the four common data mining methods in the area of pharmacovigilance are: the proportional reporting ratio (PRR) [1] used by the European Medicines Agency; the reporting odds ratio (ROR) [5] used by the Netherlands; the gamma Poisson shrinkage [6], an extension of which makes it possible to study multi-item associations (multi-item gamma Poisson shrinker) [7], used by the FDA; and the information component (IC) [8, 9] used by WHO. Although there are several excellent methods for the mining of large databases to detect ADR signals, since neither physicians nor pharmacists are aware of unknown ADRs at the time of initial drug release, their discovery is delayed and may be difficult to make timely reports to the regulatory authorities. Therefore, it is important to encourage reports of ADRs from healthcare providers to the authorities in the early stage after initial drug release. However, factors affecting the timing of signal detection after drug release have not been clarified, although there was a report that most current FAERS reporting is not affected by the issuance of FDA alerts [10].

The aim of the current study was therefore to investigate factors affecting the timing of signal detection by comparing variations in reporting time of known and unknown ADRs after initial drug release in the USA.

## Methods

Because FAERS database is anonymized by the FDA, patient records/information were anonymized and de-identified prior to analysis. Therefore we were unable to identify an individual (patient records/information) both before and after the analysis.

### Data sources

Input data for this study were taken from the FDA AERS database produced by cleaning with methods to delete the same redundant number of individual safety reports (ISRs) and number of cases, i.e., if age, gender, and onset date were different in the same number of ISRs and cases, we deleted their data because it was assumed that they were redundant. When the number of ISRs and cases was different, the data were considered redundant if they were considered to be the same based on reports to the FDA from the pharmaceutical industry which include age, gender, and onset date. The data cleaning was performed by the Japan Pharmaceutical Information Center (JAPIC). The FDA AERS database used in this study covered the period from the first quarter (Q1) of 2002 through Q4 of 2009. The data structure of the AERS is in compliance with international safety reporting guidance, ICH E2B, consisting of 7 data sets: patient demographic and administrative information (DEMO); drug/biologic information (DRUG); adverse events (REAC); patient outcomes (OUTC); report sources (RPSR); drug therapy start and end dates (THER); and indications for use/diagnosis (INDI). The adverse events in REAC are coded using preferred terms (PTs) in the Medical Dictionary for Regulatory Activities (MedDRA) terminology. Here, version 14.1 of MedDRA/Japanese version was used.

### Target ADRs with study drugs

Both known and unknown ADRs were investigated. The definition of known ADRs was that they were documented on the label at release, while that of unknown ADRs was that they were not listed on the label at release but added to a revised label after an FDA advisory. This study arbitrarily selected the following known ADRs with their associated drugs: rhabdomyolysis with rosuvastatin; malignant syndrome with aripiprazole; and hypercalcemia with teriparatide. The arbitrarily selected unknown ADRs with their associated drugs were severe liver injury with telithromycin, suicidal behavior with varenicline, and acute pancreatitis with exenatide. These ADRs all received extensive coverage in the media and were a focus of public concern.

### PTs used for ADRs

The following PTs for ADRs were used in the data mining: Rhabdomyolysis (PT 10039020); Neuroleptic malignant syndrome (PT 10029282), Hypercalcaemia (PT 10020583), Hepatic failure (PT 10019663), Acute hepatic failure (PT 10000804), Subacute hepatic failure (PT 10056956); Hepatic encephalopathy (PT 10019660), Chronic hepatic failure (PT 10057573), and Hepatic encephalopathy prophylaxis (PT 10019660) for severe liver injury; Suicidal ideation (PT 10042458) and Suicide attempt (PT 10042464) for suicidal behavior; and Pancreatitis acute (PT 10033647), Pancreatitis (PT 10033645), Pancreatitis chronic (PT 10033649), Pancreatitis necrotising (PT 10033654), and Pancreatitis haemorrhagic (PT 10033650) for acute pancreatitis.

### Data mining

We calculated the PRR, ROR, and IC every 3 months after drug release and determined the time period until signal detection as indices of signals [11–13]. Investigation of factors affecting the duration of timing of signal detection was performed using the indices of signal detection: 1) time lag from the onset of ADRs until report to the FDA; 2) for unknown ADRs, a comparison was made of the time lag from the onset until report to the FDA before and after advisory information was issued; and 3) the time period of signal detection from initial drug release when the time lag until report is assumed to be 15 days after ADR onset, because the reporting timeline to FDA requires that all adverse events that are both serious and unexpected must be submitted within 15 calendar days of initial receipt by anyone in the employ of the applicant [14]. Only reports made to the FDA more than 15 days from ADR onset were extracted, and the change in the time period of signal detection from initial drug release was determined when the time lag until report was assumed to be 15 days after ADR onset. For reports made to the FDA within 15 days from the onset of ADRs and/or the date of the onset of ADRs was omitted from the report, we used the “last observation carried forward” date.

The signal thresholds used to indicate the presence of a safety signal included “ROR – 1.96SE > 1” for the ROR [5], “PRR > 2 with χ^2^ > 4, and the presence of at least 3 case reports” for the PRR [1], and “IC – 2 SD > 0” for the IC [15, 16]. The computations were performed using SAS version 9.2 (SAS Institute, Cary, NC, USA).

## Results

Table 1 shows the label information on ADRs for each study drug. Table 2 shows the time periods of signal detection from initial drug release in the USA.

**Table 1:** Label Information on ADRs for each drug.

Drug	ADRs	US drug release	Label information at drug release	FDA-issued advisory information	Label revision
Known ADRs					
Rosuvastatin	Rhabdomyolysis	August 2003	WARNING		
Aripiprazole	Malignant syndrome	November 2002	WARNING		
Teriparatide	Hypercalcemia	November 2002	PRECAUTION		
Unknown ADRs					
Telithromycin	Severe liver injury	April 2004		January 2006	June 2006 (added to WARNING)
Varenicline	Suicidal behavior	March 2006		November 2007 May 2008	January 2008 (added to WARNING)
Exenatide	Pancreatitis	April 2005		October 2007 August 2008	January 2008 (added to PRECAUTIONS)

**Table 2:** Comparison of the time periods from initial drug release of the original signal onset time with the time period of signal detection from initial drug release when the FDA report is assumed to occur 15 days after the onset of ADRs.

	Signal index	Original signal onset time (time period from release in USA) (months)	Estimated signal onset time when the FDA report is assumed to occur within 15 days (time period from release in USA) (months)	Decrease in time period of signal detection (months)
Known ADRs				
Rhabdomyolysis with rosuvastatin	PRR signal	2004 Q1 (5–7)	2003 Q3 (–1)	4–7
	ROR signal	2004 Q1 (5–7)	2003 Q3 (–1)	4–7
	IC signal	2004 Q2 (8–10)	2003 Q3 (–1)	7–10
Malignant syndrome with aripiprazole	PRR signal	2003 Q2 (5–7)	2003 Q1 (2–4)	1–5
	ROR signal	2003 Q1 (2–4)	2003 Q1 (2–4)	—
	IC signal	2003 Q2 (5–7)	2003 Q1 (2–4)	1–5
Hypercalcemia with teriparatide	PRR signal	2003 Q3 (7–9)	2003 Q2 (5–7)	0–4
	ROR signal	2003 Q2 (5–7)	2003 Q1 (2–4)	1–5
	IC signal	2003 Q2 (5–7)	2003 Q1 (2–4)	1–5
Unknown ADRs				
Severe liver injury with telithromycin	PRR signal	2006 Q1 (21–23)	2005 Q2 (9–11)	10–14
	ROR signal	2005 Q4 (18–20)	2005 Q2 (9–11)	7–11
	IC signal	2006 Q1 (21–23)	2005 Q2 (9–11)	10–14
Suicidal behavior with varenicline	PRR signal	2007 Q4 (19–21)	2006 Q4 (7–9)	10–14
	ROR signal	2007 Q4 (19–21)	2007 Q1 (10–12)	7–11
	IC signal	2008 Q1 (22–24)	2007 Q1 (10–12)	10–14
Pancreatitis with excenatide	PRR signal	2008 Q4 (42–44)	2005 Q3 (3–5)	37–41
	ROR signal	2008 Q1 (33–35)	2005 Q2 (0–2)	31–35
	IC signal	2008 Q2 (36–38)	2005 Q2 (0–2)	34–38

### Investigation of signal detection time of known ADRs

#### Rhabdomyolysis with rosuvastatin

Rosuvastatin was released in August 2003 in the USA, and rhabdomyolysis was described as a WARNING on the label at that time (Table 1). S1 Table shows the number of reports on the use of rosuvastatin and the reporting of rhabdomyolysis as the outcome of the three data-mining algorithms with respect to the risk of rhabdomyolysis associated with the use of rosuvastatin. The total number of ADR case reports from Q3 of 2003 to Q1 of 2007 in the JAPIC-AERS was 4,237,762, of which 220 listed rhabdomyolysis and rosuvastatin (See S1 Table).

A signal of rhabdomyolysis was first detected in Q1 of 2004 using the ROR and PRR and in the second quarter (Q2) of 2004 using the IC. This period of signal detection of rhabdomyolysis was 5–7 months for both PRR and ROR and 8–10 months for IC after rosuvastatin was released in the USA (Table 2).

#### Malignant syndrome with aripiprazole

Aripiprazole was released in November 2002 in the USA, and malignant syndrome as an ADR was described as a WARNING on the label (Table 1). S2 Table shows the number of reports on the use of aripiprazole and the reporting of malignant syndrome, as measures of the three data-mining algorithms with respect to the risk of malignant syndrome associated with the use of aripiprazole. The total number of reports of ADRs in the JAPIC AERS from Q4 of 2002 to Q2 of 2006 was 3,929,114, of which 82 were reports of malignant syndrome with aripiprazole (see S2 Table).

A signal of malignant syndrome was first detected in Q1 of 2003 using the ROR and Q2 of 2003 using the PRR and IC. This period of signal detection of malignant syndrome was 2–4 months in the ROR and 5–7 months in the PRR and IC after aripiprazole release in the USA (Table 2).

#### Hypercalcemia with teriparatide

Teriparatide was released in November 2002 in the USA, and hypercalcemia was described as a PRECAUTION on the label at release (Table 1). S3 Table shows the number of reports on the use of teriparatide and the reporting of hypercalcemia, measures of the three data-mining algorithms with respect to the risk of hypercalcemia associated with the use of teriparatide. The number of reports of ADRs in the JAPIC AERS from Q2 of 2003 to Q1 of 2006 was 3,929,061, of which 58 were reports of hypercalcemia with teriparatide (see S3 Table).

A signal of hypercalcemia was first detected in Q2 of 2003 using the ROR and IC and in Q3 of 2003 using the PRR. This period of signal detection of hypercalcemia was 5–7 months in the ROR and IC and 5–7 months in the PRR after teriparatide release in the USA, (Table 2).

### Investigation of signal detection time of unknown ADRs

#### Severe liver injury with telithromycin

Telithromycin was released in April 2004 in the USA, and severe liver injury as an ADR was not listed on the label at that time (Table 1). In January 2006, the FDA issued advisory information on severe liver injury, after which the label was revised by adding severe liver injury to the WARNING section in June 2006 [17]. S4 Table shows the number of reports on the use of telithromycin and the reporting of severe liver injury, measures of the three data-mining algorithms with respect to the risk of severe liver injury associated with the use of telithromycin. The number of reports of ADRs in the JAPIC AERS from Q2 of 2004 to Q1 of 2007 was 3,494,160, of which 28 were on severe liver injury with telithromycin (See S4 Table).

A signal of severe liver injury was first detected in Q4 of 2005 using the ROR and Q1 of 2006 using the PRR and IC. This period of signal detection of severe liver injury was 18–20 months in the ROR and 21–23 months in the PRR and IC after telithromycin release in the USA (Table 2).

#### Suicidal behavior with varenicline

Varenicline was released in March 2006 in the USA, and suicidal behavior as an ADR was not listed on the label at that time (Table 1). In November 2007 and May 2008, the FDA issued advisory information on suicidal behavior [18, 19], after which the label was revised by adding suicidal behavior to the WARNING section in January 2008. S5 Table shows the number of reports on the use of varenicline and the reporting of suicidal behavior, measures of the three data-mining algorithms with respect to the risk of suicidal behavior associated with the use of varenicline. The number of reports of ADRs in the JAPIC AERS from Q1 of 2007 to Q4 of 2008 was 3,819,675, of which 123 were on suicidal behavior with varenicline (see S5 Table).

A signal of suicidal behavior was first detected in Q4 of 2007 using the ROR and PRR and Q1 of 2008 using the IC. This period of signal detection of suicidal behavior was 19–21 months in the ROR and PRR and 22–24 months in the IC after varenicline release in the USA (Table 2).

#### Pancreatitis with excenatide

Excenatide was released in April 2005 in the USA, and pancreatitis as an ADR was not listed on the label (Table 1). In October 2007 and August 2008, the FDA issued advisory information on pancreatitis, after which the label was revised by adding pancreatitis to the PRECAUTIONS in January 2008 [20, 21]. S6 Table shows the number of reports on the use of excenatide and the reporting of pancreatitis, measures of the three data-mining algorithms with respect to the risk of pancreatitis associated with the use of excenatide. The number of reports of ADRs in the JAPIC AERS from Q4 of 2005 to Q4 of 2008 was 4,762,202, of which 328 were on pancreatitis with excenatide (see S6 Table).

A signal of pancreatitis was first detected in Q1 of 2008 using the ROR, Q2 of 2008 using the IC, and Q4 of 2008 using the PRR. This period of signal detection of pancreatitis was 33–35 months in the ROR, 36–38 months in the IC, and 42–44 months in the PRR after excenatide release in the USA (Table 2).

### Investigation of time from the onset of ADRs until report to the FDA

**Fig 1:**
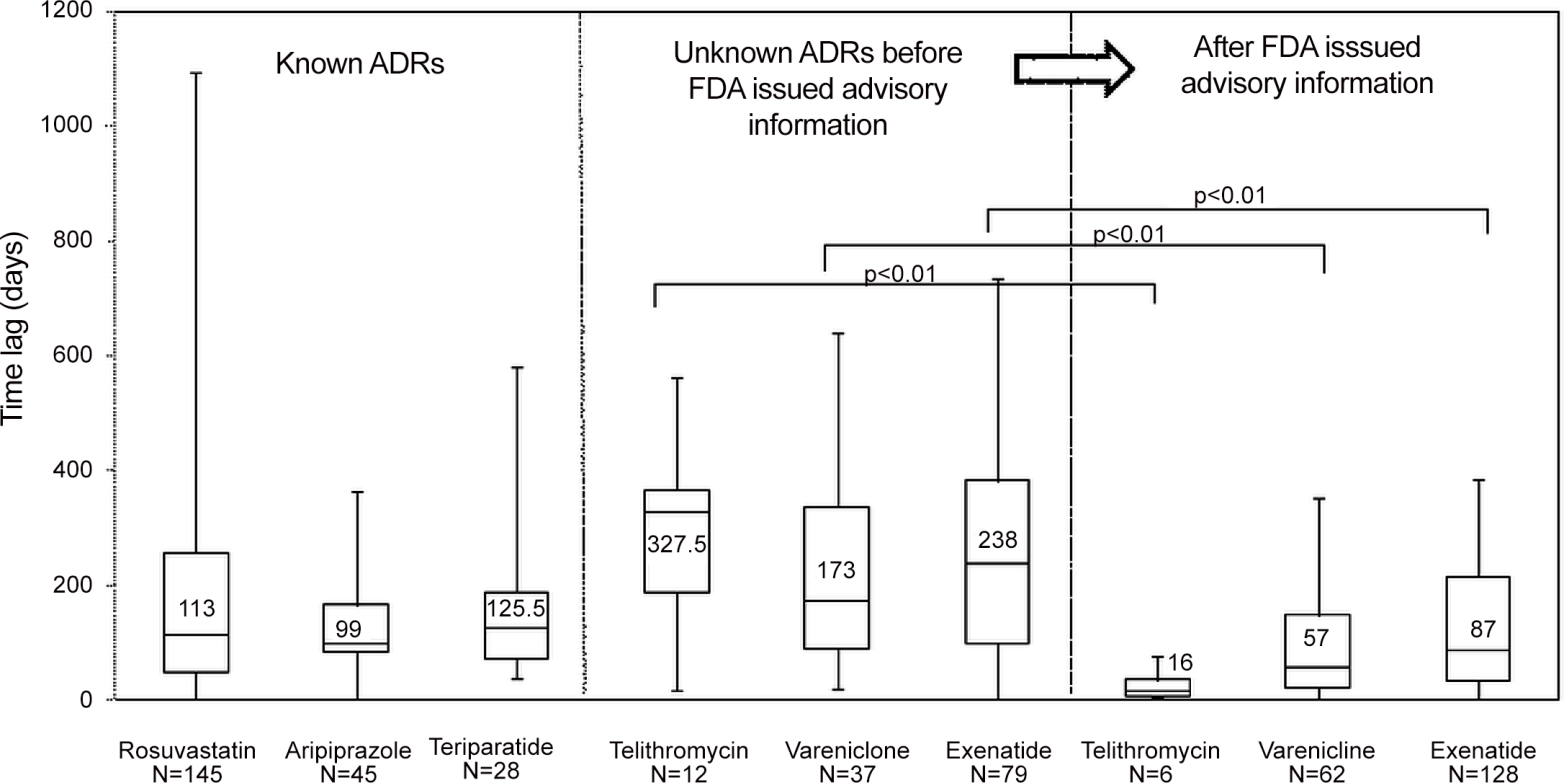
Time lag from the onset of known and unknown ADRs until report to the FDA for each study drug and effect of FDA advisory information on reporting time.

Fig 1 shows the time lag from the onset of both known and unknown ADRs until they were reported to the FDA for each drug studied. It also indicates the effect of the FDA issuing an advisory on the reporting time. Among the known ADRs, the median (25th–75th percentile) time lag from onset until report to the FDA was 113 (47–257) days for rosuvastatin, 99 (82–166) days for aripiprazole, and 125.5 (71.5–187.5) days for teriparatide. Among the unknown ADRs before the FDA issued advisory information, the median time lag from onset until report to the FDA was 327.5 (187.8–365.5) days for telithromycin, 173 (90–336) days for varenicline, and 238 (99–382) days for excenatide. After the FDA issued advisory information, the time lag was 16 (5.8–34.5) days, 57 (22–148) days (22–148), and 87 (32–214.3) days, respectively. The lag time for unknown ADRs was therefore longer than that for known ones.

After the FDA issued advisory information, the median (25th percentile–75th percentile) time lag from the onset of ADRs until report to the FDA for telithromycin, varenicline, and excenatide decreased from 327.5 (187.8–365.5) to 16 (5.8–34.5) days (p<0.01), 173 (90–336) to 57 (22–148) days (p<0.01), and 238 (99–382) to 87 (32–214.3) days (p<0.01), respectively. It is clear that, for the drugs examined here, the issuing of an FDA advisory on rare but potentially serious health risks of unknown ADRs significantly shortened the time lag from onset until report.

While the length of the time lag to report acute pancreatitis with exenatide was the second longest of the 3 drugs, the time period of signal detection was longer (33–44 months) than for the other 2 drugs (18–24 months) (Table 2).

### Investigation of time period of signal detection from initial drug release when the FDA report is assumed to occur 15 days after the onset of ADRs

Table 2 shows the results on the time period of signal detection from initial drug release when the FDA report is assumed to occur 15 days after the onset of ADRs. Time lags exceeding 15 days from the onset of known ADRs until they were reported to the FDA were found for 133 reports of rhabdomyolysis with rosuvastatin, 43 of malignant syndrome with aripiprazole, and 28 of hypercalcemia with teriparatide. For unknown ADRs, there were 16 reports of severe liver injury with telithromycin, 86 of suicidal behavior with varenicline, and 228 of pancreatitis with exenatide with time lags of greater than 15 days before reporting.

The time period of signal detection from initial drug release when the FDA report is assumed to occur 15 days after the onset of ADRs was clearly shorter. For known ADRs, it decreased to 0–1 month from 5–7 months in the PRR and ROR and from 8–10 months in the IC for rhabdomyolysis with rosuvastatin, to 2–4 months from 5–7 months in the ROR and IC and to 5–7 months from 8–10 months in the PRR for hypercalcemia with teriparatide, and to 2–4 months from 5–7 months in the PRR and IC but it did not decrease from 2–4 months in the ROR for malignant syndrome with aripiprazole. The decreases in lag time for unknown ADRs were to 9–11 months from 18–20 months in the ROR and from 21–23 months in the PRR and IC for severe liver injury with telithromycin; to 7–9 months from 19–21 months in the PRR, to 10–12 months from 19–21 months in the ROR, and from 22–24 months in the IC for malignant syndrome with aripiprazole; to 2–4 months from 5–7 months in the ROR and IC for suicidal behavior with varenicline; and to 0–2 months from 33–35 months in the ROR and from 36–38 months in the IC, and to 3–5 months from 42–44 months in the PRR for pancreatitis with exenatide (Table 2).

## Discussion

Spontaneous reporting databases are useful for detecting rare, serious ADRs. Recently, collecting direct ADR reports from patients has come to be regarded as important, and approximately 50 countries have direct spontaneous reporting systems for patients [22]. The early detection of possible ADRs is one result of data mining of large databases of spontaneous reporting systems. There are many data mining reports to detect rare and/or unknown ADRs using spontaneous ADR reporting systems [2], and therefore spontaneous ADRs reporting system serve as a powerful pharmacovigilance tool. This requires the proactive reporting by as many healthcare professionals as possible, as well as an understanding of factors that affect the decision to report a potential ADR.

In recent years, in addition to detecting rare and/or unknown ADRs, spontaneous ADR reporting systems have been used to identify hidden drug–drug interactions [3], predict potential drug targets [4], and determine the extent of stimulated reporting in response to warnings, alerts, and label change in adverse event reports [10]. However, there are few studies on the factors affecting the timing of signal detection by comparing variations in reporting times of known and unknown ADRs after initial drug release in the USA.

We randomly selected 3 drugs for both known and unknown ADRs in this study. However, ADRs such as carcinoma that take a long time to develop from the start of drug administration were excluded from our analyses.

The time period for the detection of signals after drug release in the USA was 2–10 months for known ADRs and 18–44 months for unknown ones. The median lag time from onset until reporting for known and unknown ADRs was 99.0–125.5 days and 173.0–327.5 days, respectively (Table 2, Fig 1). Regardless of the index of signal detection used, both the time period for the detection of a signal after drug release in the USA and the median lag time from onset until reporting were shorter for known than for unknown ADRs. The reason for delayed signal detection is believed to be that it is difficult to determine an association between a drug and an unknown ADR, and therefore a longer time is required until that ADR is recognized. Compared with known ADRs, unknown ones tend to be underreported, thereby lengthening the time lag from onset to FDA report. After the FDA issued an advisory on rare but potentially serious health risks of unknown ADRs, the time lag from onset to reporting decreased (Fig 1). Therefore, the difference in the time lag between reporting known and unknown ADRs is considered to be due to whether an ADR was previously known or unknown. If unknown ADRs were reported more rapidly, earlier label revisions could be made.

Hauben *et al*. [23] reported a marked increase in the number of spontaneous reports of hyperkalemia with spironolactone and the PRR EB05 since the publication of the RALE study, which indicated that an increased risk of serious hypercalemia occurred with the combination of spironolactone and ACE inhibitors. These results suggest as a specific ADR becomes well known, the number of spontaneous reports and its signal increase. This appears to be an example of the “bandwagon phenomenon” made famous by the mass media, in which the number of ADRs increases rapidly [24].

For telithromycin, 22 of 28 cases of severe liver injury with telithromycin were reported from Q1 2006 (S4 Table). Three case reports on severe liver injury with telithromycin were published on January 20, 2006. Subsequently, the FDA started a study of the causal association between severe liver injury and telithromycin, and based on the results the telithromycin label was revised in June 2006. It is thus possible that those case reports prompted the FDA to investigate the drugs more thoroughly, leading to the confirmation of the causal association between ADRs and the drugs. Also, 121 of 123 cases of suicidal behavior with varenicline were reported from Q4 2007 (S5 Table), and 284 of 328 cases of pancreatitis with exenatide from Q4 2007 (S6 Table). We believe that these increases in ADR numbers after the FDA issued alerts reflect the bandwagon phenomenon.

This study found gaps occurring among the signal detection times when using PRR, ROR, and IC as signal indices. In rhabdomyolysis with rosuvastatin and suicidal behavior with varenicline, the signal detection time of the ROR and PRR was the shortest, followed by the IC. In malignant syndrome with aripiprazole and severe liver injury with telithromycin, the signal detection time of the ROR was the shortest, followed by simultaneous signal detection with the PRR and IC. In hypercalcemia with teriparatide, the ROR and IC showed the shortest signal detection times, followed by the PRR. In pancreatitis with excenatide, the signal detection time was in the order ROR, IC, and PRR. These differences are assumed to result from the sensitivity of signals. According to one report on data-mining techniques [25], the sensitivity of signal detection when mining databases using the AERS was 47.6% in the ROR, 35.2% in the PRR, and 34.3% in the IC. That result agrees with the detection rates of signals in our study and indicates that the differences in signal sensitivity influence the time of signal detection. The previous report [26] also mentioned that there is a trade-off relationship between sensitivity and specificity: if the time of signal detection is earlier, its signal index may not be superior. It is recommended that signal detection should be performed in future using several signals in combination after clarifying the characteristics of each index.

The results of this study suggested that whether an ADR is known or unknown is a factor that may influence the time of signal detection, and that the time from ADR onset to FDA report is longer for unknown ADRs. If ADRs are reported promptly to the FDA, it is likely that their signals will be detected early. It is difficult to pinpoint a causal association between a drug and an unknown ADR, and therefore it is probable that no report will be made to the FDA even if an ADR occurs. All healthcare professionals and consumers need to report to the FDA proactively when ADRs are recognized, even if the association with a drug is unknown. We believe that this reporting by healthcare professionals and consumers could lead to the issuance of US FDA alerts, although Hoffman suggested that most current FAERS reporting is not significantly affected by the issuance of FDA alerts except for certain drugs [10].

In this study, we counted the number of reports in a combined database because it provided a convenient means of data analysis. One study found that no major difference in sensitivity and specificity, i.e., no inferior-to-superior difference, was found when using different methods to count the number of reports of specific ADRs compared with the total number of ADR reports [23]. However, this needs further study, because it is possible that the results could differ depending on the total number of reports in the database. In future, we will attempt to confirm the hypothesis suggested in this study that the difficulty in identifying a causal association between an unknown ADR and a drug may result in underreporting to the FDA by increasing the number of drugs studied. It will also be necessary to find a method to resolve this problem.

## Conclusion

The present results suggest that one factor affecting the signal detection time is whether the ADR is known or unknown before its occurrence. To detect the signals of unknown ADRs more rapidly and/or prevent critical ADR events, healthcare professionals and consumers should make reports to the FDA proactively even if the association between a drug and ADR is not confirmed.

## Supporting Information

S1 Table Number of reports on the use of rosuvastatin and the reporting of rhabdomyolysis and measures of the three data-mining algorithms with respect to the risk of rhabdomyolysis associated with the use of rosuvastatin. Na: Number of reports on the use of rosuvastatin and the reporting of rhabdomyolysis. Nb: Number of reports on the use of other drugs and the reporting of rhabdomyolysis. Nc: Number of reports on the use of rosuvastatin and the reporting of other adverse events. Nd: Number of reports on the use of other drugs and the reporting of other adverse events.
(DOCX)

S2 Table Number of reports on the use of aripiprazole and the reporting of malignant syndrome and measures of the three data-mining algorithms with respect to the risk of malignant syndrome associated with the use of aripiprazole. Na: Number of reports on the use of aripiprazole and the reporting of malignant syndrome. Nb: Number of reports on the use of other drugs and the reporting of malignant syndrome. Nc: Number of reports on the use of aripiprazole and the reporting of other adverse events. Nd: Number of reports on the use of other drugs and the reporting of other adverse events.
(DOCX)

S3 Table Number of reports on the use of teriparatide and the reporting of hypercalcemia and measures of the three data-mining algorithms with respect to the risk of hypercalcemia associated with the use of teriparatide. Na: Number of reports on the use of teriparatide and the reporting of hypercalcemia. Nb: Number of reports on the use of other drugs and the reporting of hypercalcemia. Nc: Number of reports on the use of teriparatide and the reporting of other adverse events. Nd: Number of reports on the use of other drugs and the reporting of other adverse events.
(DOCX)

S4 Table Number of reports regarding the use of telithromycin and the reporting of severe liver injury and measures of the three data-mining algorithms with respect to the risk of severe liver injury associated with the use of telithromycin. Na: Number of reports with the use of telithromycin and the reporting of severe liver injury. Nb: Number of reports with the use of other drugs and the reporting of severe liver injury. Nc: Number of reports with the use of telithromycin and the reporting of other adverse events. Nd: Number of reports with the use of other drugs and the reporting of other adverse events.
(DOCX)

S5 Table Number of reports on the use of varenicline and the reporting of suicidal behavior and measures of the three data-mining algorithms with respect to the risk of suicidal behavior associated with the use of varenicline. Na: Number of reports on the use of varenicline and the reporting of suicidal behavior. Nb: Number of reports on the use of other drugs and the reporting of suicidal behavior. Nc: Number of reports on the use of varenicline and the reporting of other adverse events. Nd: Number of reports on the use of other drugs and the reporting of other adverse events.
(DOCX)

S6 Table Number of reports on the use of excenatide and the reporting of pancreatitis and measures of the three data-mining algorithms with respect to the risk of pancreatitis associated with the use of excenatide. Na: Number of reports on the use of excenatide and the reporting of pancreatitis. Nb: Number of reports on the use of other drugs and the reporting of pancreatitis. Nc: Number of reports on the use of excenatide and the reporting of other adverse events. Nd: Number of reports on the use of other drugs and the reporting of other adverse events.
(DOCX)
