# Supplementary material for: Factors Affecting the Timing of Signal Detection of Adverse Drug Reactions
Source: PLoS One. 2015 Dec 7;10(12):e0144263. doi: 10.1371/journal.pone.0144263 (PMC4671724; doi:10.1371/journal.pone.0144263)
Supplement: S6 Table — Na: Number of reports on the use of excenatide and the reporting of pancreatitis. Nb: Number of reports on the use of other drugs and the reporting of pancreatitis. Nc: Number of reports on the use of excenatide and the reporting of other adverse events. Nd: Number of reports on the use of other drugs and the reporting of other adverse events. (DOCX) [file pone.0144263.s006.docx]

S6 Table
